# Implementing the Lolli-Method and pooled RT-qPCR testing for SARS-CoV-2 surveillance in schools: a pilot project

**DOI:** 10.1007/s15010-022-01865-0

**Published:** 2022-06-27

**Authors:** Alina Chloé Kretschmer, Lena Junker, Felix Dewald, Viktoria Linne, Lea Hennen, Gibran Horemheb-Rubio, Rolf Kaiser, Gertrud Steger, Alexander Joachim, Jana Schönenkorb, Zülfü Cem Cosgun, Neslihan Mühlhans, Eva Heger, Elena Knops, Charlotte Leisse, Barbora Kessel, Torben Heinsohn, Isti Rodiah, Berit Lange, Anne Lena Ritter, Mira Fries, Annelene Kossow, Johannes Nießen, Jörg Dötsch, Florian Klein, Jan Rybniker, Gerd Fätkenheuer, Isabelle Suárez

**Affiliations:** 1 Department I of Internal Medicine, Division of Infectious Diseases, University Hospital Cologne, University of Cologne, Kerpener Str. 62, 50937 Cologne, Germany; 2 German Center for Infection Research (DZIF), Partner Site Bonn-Cologne, Cologne, Germany; 3 Center for Molecular Medicine Cologne, University of Cologne, 50931 Cologne, Germany; 4 Institute of Virology, University Hospital Cologne, University of Cologne, Cologne, Germany; 5 Department of Pediatrics, University Hospital Cologne, University of Cologne, Cologne, Germany; 6 Public Health Department Cologne, Cologne, Germany; 7 School Department Cologne, Cologne, Germany; 8 Department of Epidemiology, Helmholtz Centre for Infection Research, Brunswick, Germany; 9 German Centre for Infection Research (DZIF), TI BBD, Brunswick, Germany; 10 Federal Ministry of Education and Research (BMBF), B-FAST Project in “NaFoUniMedCovid19” (FKZ: 01KX2021), Bonn, Germany; 11 Institute of Hygiene, University Hospital Muenster, Muenster, Germany

**Keywords:** COVID-19, SARS-CoV-2, School, Pooled testing, Lolli-Method, RT-qPCR

## Abstract

**Purpose:**

School closures have been used as part of lockdown strategies to contain the spread of SARS-CoV-2, adversely affecting children’s health and education. To ensure the accessibility of educational institutions without exposing society to the risk of increased transmissions, it is essential to establish SARS-CoV-2 testing strategies that are child-friendly, scalable and implementable in a daily school routine. Self-sampling using non-invasive saliva swabs combined with pooled RT-qPCR testing (Lolli-Method) has been proven to be a sensitive method for the detection of SARS-CoV-2.

**Methods:**

We conducted a pilot project in Cologne, Germany, designed to determine the feasibility of a large-scale rollout of the Lolli-Method for testing without any additional on-site medical staff in schools. Over a period of three weeks, students from 22 schools were sampled using the Lolli-Method. At the end of the project, teachers were asked to evaluate the overall acceptance of the project.

**Results:**

We analyzed a total of 757 pooled RT-qPCRs obtained from 8,287 individual swabs and detected 7 SARS-CoV-2 infected individuals. The Lolli-Method was shown to be a feasible and accepted testing strategy whose application is only slightly disruptive to the daily school routine.

**Conclusion:**

Our observations suggest that the Lolli-Method in combination with pooled RT-qPCR can be implemented for SARS-CoV-2 surveillance in daily school routine, applicable on a large scale.

**Supplementary Information:**

The online version contains supplementary material available at 10.1007/s15010-022-01865-0.

## Introduction

The SARS-CoV-2 pandemic implies major challenges for our society and has a strong impact on various aspects of social life [1, 2]. Children and adolescents have been markedly affected by school closures, ranging from impacts on education to mental health [3, 4]. Closing educational institutions is likely to exacerbate social disparities, as children from less advantaged backgrounds tend to be most affected [5]. Therefore, keeping schools open is of utmost concern [6].

Children often show mild courses of SARS-CoV-2 infection or may even present without symptoms [7]. However, they still may be infectious with high viral loads [8]. Furthermore, recent observations indicate a shift of infection pattern toward the younger unvaccinated population, as SARS-CoV-2 vaccines have only recently been licensed for children between 5 and 11 years of age [9]. Thus, systematic testing of the general, asymptomatic population was proposed early in the pandemic for limiting transmissions [10] and needs to be established in schools for surveillance and infection control. However, conducting regular testing poses a substantial challenge to laboratory capacities [10].

From November to December 2020, the multicentre intervention study “Bundesweites Forschungsnetz Angewandte Surveillance und Testung” (B-FAST) was initiated with the intention of developing comprehensive and scalable surveillance strategies. Within this controlled randomized study with 3,970 participants [11], we implemented the Lolli-Method, a non-invasive self-sampling method for saliva samples, combined with RT-qPCR pooled testing as a highly sensitive and broadly accepted method for SARS-CoV-2 screening at educational institutions. The diagnostic sensitivity of the Lolli-Method was shown to be 93.9% when the viral load of corresponding Np-/Op-swabs was > 10^3^ copies/ml^12^. Sample collection consists of sucking on a swab (like a lollipop). The swabs of the entire class are jointly being analyzed for SARS-CoV-2 by pooled RT-qPCR in the laboratory. However, the presence of medical staff for supervision of the sample collection was mandatory during the study period for regulatory reasons. Thus, it could not be determined whether the method also proves to be applicable in everyday school life without the additional support of medical staff. This pilot project aimed at determining the feasibility of the Lolli-Method, implementable in all types of schools.

## Methods

### Implementation

During three weeks in March 2021 (commencing 8th of March), students from 22 (8%) out of 285 schools in the city of Cologne participated in the project. Different types of schools, ranging from elementary to secondary and special needs schools, in nine socially heterogeneous city districts, were selected to be representative for the educational and social diversity in Cologne. When signing up for the project, schools were asked to estimate the number of students participating in the project, so that the supply logistics as well as testing capacities could be determined. When participating, students or their legal guardians had to provide informed written consent prior to the start of testing. Each participant was tested at least once a week. At that time, students from elementary schools attended class in alternation, resulting in only half of each class present a day. In secondary schools, only the graduating classes were present.

As a result, there were two test days a week in primary and special needs schools and one test day a week in secondary schools. The schools were supplied with test material (swabs, tubes, transport bags, labels, etc.) the week prior the start of testing.

To enable non-medical school staff to perform the sample collection with their students successfully as well as having a full understanding of the project, representatives of every participating school were trained in a video conference by a team of the University Hospital of Cologne. In addition, the participating schools received detailed written instructions explaining how to collect the samples, how to label them for transport as well as notification procedures in case of a positive pool. A website containing information tailored to the target groups (teachers, parents and students) and a short explanatory video, was launched.

During the testing period, a telephone hotline (9 am–12 pm, Monday to Friday) was available to solve problems and answer arising questions. Each week, a short newsletter containing project updates was sent to the participating schools.

### Sample collection applying the Lolli-Method

The sample collection routinely took place in school before class started. Samples were taken with a standard dry swab (polystyrol sticks with viscose tip without medium). The sealed swabs were distributed to the attending students. Under guidance of the teacher, all students of a class simultaneously took saliva samples using the Lolli-Method by 30 s sucking on a swab. The students performed the sample collection on their own. The swabs were then collected in 50 ml centrifugation tubes labeled with the class and school names. In addition, an individual saliva sample of each participating student was collected separately. This was only to be analyzed if the pooled test turned out to be positive for SARS-CoV-2 to identify the infected individual. Shortly after collection, samples were picked up by a laboratory specimen transport and delivered to the Institute of Virology of the University Hospital of Cologne. Upon arrival the samples were immediately analyzed using RT-qPCR pooled testing. Further laboratory procedures are described in the supplement.

### Reporting procedure

In case of a positive RT-qPCR pooled test, the headmaster of the affected school was notified on the same day, so that families could be informed. Students of the respective pooled sample had to remain in quarantine for the next day until the SARS-CoV-2-positive individual from the pool was identified. The Public Health Department of Cologne was notified subsequently. SARS-CoV-2-positive students and close contact persons determined by the Public Health Department had to remain in quarantine, whereas the rest of the participating students were allowed to continue attending school. SARS-CoV-2-negative test results were transmitted via e-mail to the schools over-night.

### Data analysis

For this retrospective analysis only anonymized, de-identifiable data were used. Data processing and statistical analysis were performed using the software GraphPad Prism Version 8.0. Student characteristics were reported as absolute numbers with percentage. We assessed the number of conducted tests, the number of pools, the number of SARS-CoV-2-positive RT-qPCR pooled tests, and the number of SARS-CoV-2 positive individuals detected by the Lolli-Method. To assess the feasibility of the test strategy, we analyzed the data derived from an online survey that teachers were asked to complete at the end of the 3-week test phase, evaluating the duration of sample collection, the interruption of class due to lolli tests, and the suitability of the Lolli-Method for school screening from a teacher’s perspective.

### Ethical considerations

This retrospective analysis was approved by the ethics committee of the medical faculty, University of Cologne (registration number 21-1358). Participation was voluntary. Participating students or their legal guardians had to provide informed written consent prior to the start of testing. The project complied with EU and German data protection regulations.

## Results

### Feasibility of logistics and testing

In terms of logistics, the delivery of materials was successful and on schedule. School staff was able to prepare the material (e.g., swabs) using the provided test kits without any problems. The sample collection for each class was mostly completed in time for sample pick-up. Only in three cases, samples were submitted late to the laboratory, but could still be analyzed as intended.

Sample collection was performed by each student registered for testing and present in school on the day of testing. The collection of swabs by teaching staff was successful. However, in the first week of testing, two common errors arose which resulted in some samples not being able to be processed by the laboratory: incomplete, wrongly and illegibly labeled samples as well as falsely loaded centrifugation tubes. Those errors could be eliminated after the first week by contacting the respective school staff.

### Test results

In a total of 757 pooled RT-qPCR analyses obtained from 8,287 (week 1: 2,087, week 2: 3,090, week 2: 3,110) individual swabs (Table 1), we identified seven positive pooled tests (1% of pooled analyses). RT-qPCR analyses performed with non-pooled back-up samples of the students from pool-positive classes also revealed seven SARS-CoV-2-positive individuals. All SARS-CoV-2-positive students were asymptomatic. These SARS-CoV-2-positive individuals were students from elementary schools, five of them were female, two were male. Pools contained an average of 12 saliva swabs. In six out of seven cases, the results were reported to the schools on the same day. In one class with a positive pooled test, all analyzed individual samples remained negative. In consequence, all students belonging to the pool underwent re-testing applying the Lolli-Method with individual samples the next morning, which then identified the SARS-CoV-2-positive individual.

**Table 1:** Overview of participating schools and identified SARS-CoV-2-positive cases

	Week 1	Week 2	Week 3	Total
Primary and special schools (*n* = 15)				
Pools	169	211	213	593
Individual swabs	1591	2293	2292	6176
Positive pools	2 (1.2%)	1 (0.47%)	4 (1.88%)	7 (1.18%)
Secondary schools (*n* = 7)				
Pools	40	62	62	164
Individual swabs	487	797	818	2102
Positive Pools	0	0	0	0
Total (*n* = 22)				
Pools	209	273	275	757
Individual swabs	2087	3090	3110	8287
Positive pools	2 (1%)	1 (0.3%)	4 (1.45%)	7 (0.92%)

### Online survey for teachers evaluating the test method

**Fig. 1:**
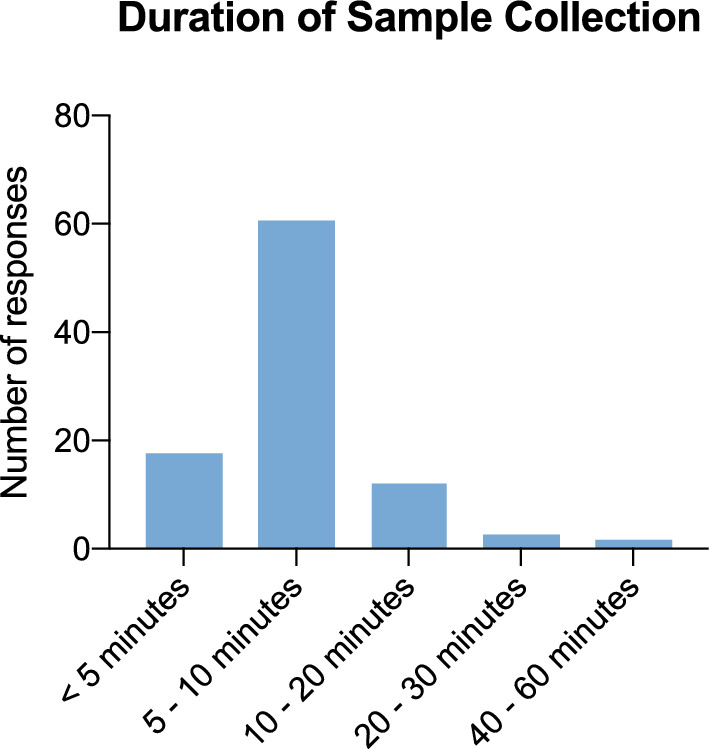
Duration of Sample Collection Duration of Sample Collection rated by teachers who filled in the online questionnaire (*n* = 96)

**Fig. 2:**
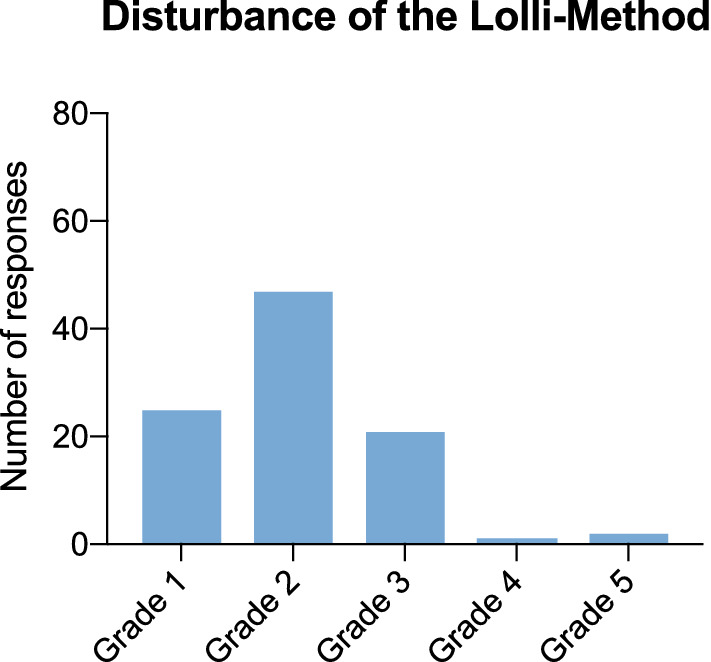
Disturbance of the Lolli-Method Evaluation of the disturbance of the Lolli-Method during the class by grading (Grade 1 = not disturbing, Grade 5 = very disturbing)

**Fig. 3:**
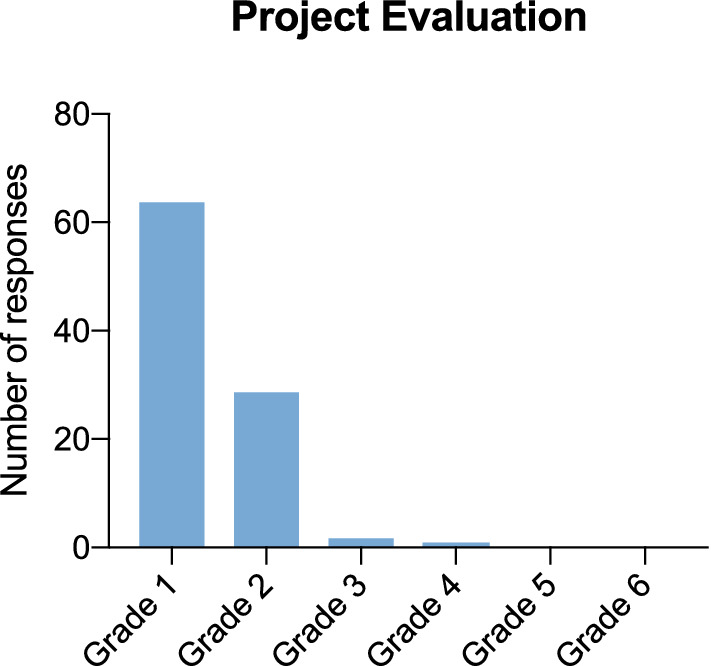
Project Evaluation Evaluation of the pilot project by grading (Grade 1 = not disturbing, Grade 5 = very disturbing)

96 teachers participated in the online survey (69 from primary schools, 23 from special need schools, 4 from secondary schools). Two-thirds of the teachers (64%, *n* = 61) reported that the sampling time lasted between 5 and 10 min (Fig. 1). Further, 76% of teachers (*n* = 73) indicated, that the duration decreased after the first test day, 24% (n = 23) found no change. The Lolli-Method was not considered as disruptive in terms of teaching by 26% (*n* = 25), while 49% (*n* = 47) considered it slightly disruptive. Only 2% (*n* = 2) stated that performing the lolli tests was very disruptive (Fig. 2). In addition, 92% (*n* = 88) found the Lolli-Method more suitable for the everyday use in schools compared to the use of rapid antigen tests. The overall project was rated outstanding by 97% (*n *= 93) (Fig. 3).

## Discussion

With this pilot project, we present a feasible and accepted testing strategy, suitable for everyday use in educational institutions without requiring additional on-site medical staff. The Lolli-Method is a child-friendly and safe method of sampling. Students, also those of young age, are able to perform sampling independently without assistance. Surveillance strategies using pooled samples have been previously described in study settings leading to a scalable and resource-efficient screening tool [13–16]. It allows the simultaneous sample collection of up to 30 individual swabs (e.g., an entire school class), providing a time and cost-effective testing method for SARS-CoV-2 surveillance [11] in educational institutions. During the project period, we detected seven positive pooled tests (0.9%), identifying seven SARS-CoV-2-positive students from elementary schools. As our project was performed in March 2021 where incidence rates of SARS-CoV-2 were decreasing in Germany, the low rate of case detection is not surprising [17, 18] (Table 1). In most schools, the testing was carried out only once a week per student. To minimize the number of missed infections, it is conceivable that testing should be performed more often [12].

The aim of the present project was to assess the feasibility of the surveillance program based on the Lolli-Method and RT-qPCR pool analysis in terms of acceptance and logistics. Even though the initial set-up appears to be relatively complex, since test days have to be determined for each school, routes for the laboratory specimen transport have to be planned and test material has to be delivered, we hereby prove feasibility once the structures are established. Results of our online survey indicate, that the Lolli-Method can easily be integrated in everyday school life without disrupting the teaching schedule (Fig. 3). Sweeney-Reed et al. assessed the acceptance of gargle samples taken at home combined with pooled tests [19]. When asked about the preferred location for sample collection, most participants preferred sample collection at school. One reason could be, that the presence of teachers ensures the test is carried out correctly.

Within this pilot project, an individual lolli swab of each participating student was collected, but only analyzed if the pooled test turned out to be positive for SARS-CoV-2. This approach not only has the disadvantage that many swabs had to be discarded, but also that it may not be feasible in case of supply shortages, which have been common during the pandemic. An alternative, and thus more resource-saving option, would be to perform a single test on the following day in case of a positive pooled test to identify the SARS-CoV-2 positive individual. The result of a positive pooled test, as well as the result of the subsequent analyzed individual sample, was available on average 6–8 h after the samples had reached the laboratory. Thus, respective schools were notified on the same day to prevent students from a SARS-CoV-2-positive pool coming to school the following day.

An alternative, widely used method for regular screening is based on rapid antigen detection tests (RADTs) [20, 21]. In contrast to the Lolli-Method, RADTs provide immediate results. Arguably, the delay of provided results in the RT-qRCR-pooled tests may be seen as a limitation of this screening method as potentially infectious students remain at school. On the other hand, RT-qPCR-pool testing reduces stigmatization as a student’s positive tests results are not immediately disclosed to their classmates leading to an increased acceptance of this surveillance method. In addition, we have recently compared the sensitivity of RADTs with that of the Lolli-Method obtained by RT-qPCR pool tests [12, 22, 23]. The Lolli-Method has proven to be more sensitive and therefore has the potential to detect SARS-CoV-2-positive individuals before they become highly infectious [12, 22]. Furthermore, handling of RADTs appears to be more difficult than the Lolli-Method, especially for young children that may need help from school staff.

Some errors occurred during sample collection that partly hindered correct processing of the samples. This may be attributable to lack of experience of teaching staff in handling of medical equipment or limited understanding of the procedure. However, those difficulties were fully eliminated after the first week of testing, demonstrating that the sample collection using the Lolli-Method can be conducted by non-medical staff with some practice. In addition, uncertainties in labeling samples may be avoided using pre-labeled testing material. Providing sufficient information and timely test results is key to build trust in the project among teachers as well as students and their families. Ultimately, a medical procedure is transferred to a school setting.

We were able to demonstrate that the Lolli-Method as a screening strategy for SARS-CoV-2 in the daily school routine is feasible and can be applicable on a large scale. Based on the experience gained from the pilot project, the test concept was implemented as a SARS-CoV-2 screening program at elementary schools and special needs schools in North-Rhine Westphalia [12]

## Supplementary Information

Below is the link to the electronic supplementary material.

**Supplementary file:1** (DOCX 28 KB)
