# Supplementary material for: Implementing the Lolli-Method and pooled RT-qPCR testing for SARS-CoV-2 surveillance in schools: a pilot project
Source: Infection. 2022 Jun 27;51(2):459–64. doi: 10.1007/s15010-022-01865-0 (PMC9243733; doi:10.1007/s15010-022-01865-0)
Supplement: Supplementary file 1 — Supplementary file:1 (DOCX 28 KB) [file 15010_2022_1865_MOESM1_ESM.docx]

**Supplementary appendix for:**

**Implementing the Lolli-method and pooled RT-qPCR-Testing for SARS-CoV-2 surveillance in schools: a pilot project**

Alina Chloé Kretschmer^1^, Lena Junker^1^, Felix Dewald^4^, Viktoria Linne^1,2^, Lea Hennen^1^, Gibran Horemheb-Rubio^4^, Rolf Kaiser^4^, Gertrud Steger^4^, Alexander Joachim^5^, Jana Schönenkorb^5^, Zülfü Cem Cosgun^5^, Neslihan Mühlhans^4^, Eva Heger^4^, Elena Knops^4^, Charlotte Leisse^1^, Barbora Kessel^8^, Torben Heinsohn^8^, Isti Rodiah^8^, Berit Lange^8,9^, Anne Lena Ritter^7^, Mira Fries^6^, Annelene Kossow^6^, Johannes Nießen^6^, Jörg Dötsch^5^, Florian Klein^3,4^, Jan Rybniker^1,2,3^, Gerd Fätkenheuer^1,2^, Isabelle Suárez^1,2^

1) Department I of Internal Medicine, Division of Infectious Diseases, University Hospital Cologne, University of Cologne, Cologne, Germany

2) German Center for Infection Research (DZIF), Partner Site Bonn-Cologne, Cologne, Germany

3) Center for Molecular Medicine Cologne, University of Cologne, 50931 Cologne, Germany

4) Institute of Virology, University Hospital Cologne, University of Cologne, Cologne, Germany

5) Department of Pediatrics, University Hospital Cologne, University of Cologne, Cologne, Germany

6) Public Health Department Cologne, Cologne, Germany

7) School Department Cologne, Cologne, Germany

8) Department of Epidemiology, Helmholtz Centre for Infection Research, Braunschweig, Germany

9) German Centre for Infection Research (DZIF), TI BBD, Braunschweig, Germany

**Corresponding authors:**

Isabelle Suárez, M.D.

Department I of Internal Medicine
University Hospital of Cologne

Kerpener Str. 62

50937 Cologne, Germany

Tel.: 0049-221 478-0

Email: isabelle.suarez@uk-koeln.de

**Keywords:** COVID-19; SARS-CoV-2; school; pooled testing; Lolli-Method; RT-qPCR

**This document includes supplemental methods:**

**COVID-19 preventive measures in schools during project period**

Face masks for staff were mandatory at all times; in primary schools, face masks for children were mandatory when not seated at their desks. In secondary schools, face masks for students were mandatory at all times. Classes were split to reduce the number of students per classroom in primary schools, resulting in students attending in alternation.

**Collection of the Lolli-tests for pooled testing**

Before the test phase enrolled, all participating schools received written instructions containing information on handling and labelling the samples.

The Lolli-tests were collected in the morning before class started with a standard dry swab (polystyrol sticks with viscose tip in single tube without medium, no intended breaking point).

The teachers distributed two sealed swabs among the participating students. One swab sealed in plastic for the pool sample and one swab in a test tube for the individual sample. The students performed the sample collection simultaneously on their own by removing their face mask slightly and sucking on each swab for 30 seconds until the swabs were completely soaked with saliva.

The pool sample swabs were collected in a 50 mL centrifugation tube without transport medium, which was labelled with the school name and class details. The individual swabs were put back into single test tubes, which were labelled with the student’s name and date of birth. All samples of each class were collected in a polyethylene transport sample bag, which was again labelled with the school name and class details as well.

The samples were dropped off at a central point on the school ground, where they were then collected by a laboratory specimen transport between 11am-1pm on the respective test day. The samples were delivered to the Institute of Virology of the University Hospital of Cologne, the samples reached the laboratories at 2 pm latest.

**Pool processing and testing**

All samples were analyzed on the same day of sample collection in the laboratory of the Institute of Virology of the University Hospital of Cologne, to ensure that the results were available on the same day. One 50mL centrifugation tube contained up to 30 Lolli-tests and was filled with 3 mL of phosphate buffered saline (PBS) and vortexed afterwards for 30 seconds. After vortexing, 0.5 mL of PBS was used for SARS-CoV-2 RNA detection by quantitative reverse transcription-polymerase chain reaction (RT-qPCR), the rest was stored as a back-up sample. The SARS-CoV-2 RNA detection was performed using highly sensitive and commercially available kits for RT-qPCR; Cobas^®^ SARS-CoV-2 test (Roche), Alinity m SARS-COV-2 assay (Abbott), Aptima® SARS-CoV-2 assay (Hologic Panther Fusion), according to the site availability. The method was validated and sample collection was supervised by trained personnel. Results were communicated on the same day.

**Processing of individual tests in case of a positive pool**

The individual Lolli-tests were only analyzed when the respective pool turned out to be SARS-CoV-2 positive. In this case, each individual swab of the pool tested positive was placed into a 5 mL tube, pre-filled with 2 mL PBS and vortexed for 30 seconds. 0.5 mL PBS was used to test for SARS-CoV-2 RNA by RT-qPCR and the rest was stored.

**Reporting procedure in case of a positive pool**

In case of a SARS-CoV-2 positively tested pool, the result was communicated by the Institute of Virology of the University Hospital of Cologne to the school concerned on the same day. The school consecutively informed all students of the pool concerned. All students of the respective pool had to stay at home in quarantine the next day until the SARS-CoV-2 positive individual of the pool was successfully identified. The results were also transmitted to the Health Department of Cologne via a designated email address. The health department then performed contact tracing and identified the students who needed to stay in quarantine, whereas the other students could return to class.
